# Impacts of Diabetes Mellitus on Cardiovascular Outcomes and Differential Effects of Direct Oral Anticoagulants in Patients with Left Ventricular Thrombus

**DOI:** 10.31083/j.rcm2403065

**Published:** 2023-02-22

**Authors:** Boqun Shi, Rui Zhang, Chenxi Song, Kongyong Cui, Dong Zhang, Lei Jia, Dong Yin, Hongjian Wang, Ke-Fei Dou, Weihua Song

**Affiliations:** ^1^Cardiometabolic Medicine Center, Department of Cardiology, Fuwai Hospital, Chinese Academy of Medical Sciences & Peking Union Medical College/National Center for Cardiovascular Diseases, 10037 Beijing, China; ^2^Coronary Heart Disease Center, Department of Cardiology, Fuwai Hospital, Chinese Academy of Medical Sciences & Peking Union Medical College/National Center for Cardiovascular Diseases, 10037 Beijing, China; ^3^State Key Laboratory of Cardiovascular Disease, 10037 Beijing, China

**Keywords:** anticoagulation, heart failure, thrombosis, type 2 diabetes

## Abstract

**Background:**

The focus of this investigation into the impact of type 2 diabetes mellitus (T2DM) on left ventricular thrombus (LVT) is (a) the differences in LVT characteristics, (b) long-term clinical outcomes, and (c) differential effects of direct oral anticoagulants (DOAC) among patients with T2DM and without diabetes.

**Methods:**

Patients with confirmed LVT from 2009 to 2021 were included. The primary endpoints were major adverse cardiac and cerebrovascular events (MACCE), composite of cardiovascular death, ischemic stroke, and acute myocardial infarction (AMI). The secondary endpoints were all-cause death and cardiovascular death. Multivariable competing-risk regression and cumulative incidence functions (CIF) were used to evaluate the adverse consequences.

**Results:**

In total, 1675 patients were assessed initially. Follow-up data were available for 91.1% of the participants. Median follow-up was 3.8 years. This retrospective study ultimately comprised 1068 participants, of which 429 had T2DM. Significantly higher proportions of comorbidities were observed in the T2DM group. The location, morphology, and size of LVT were similar in the two groups. Multivariable analysis suggested a higher risk of MACCE among patients with T2DM. The difference in risk between the two groups after matching and weighting was not statistically significant. Among the whole sample (*n *= 638) or the just the non-diabetic patients with LVT and anticoagulation (*n *= 382), the incidence of MACCE did not differ between DOAC treatment and warfarin treatment. In the diabetic LVT population with anticoagulation (*n *= 256), DOAC treatment was associated with a significantly higher risk of MACCE than was warfarin treatment.

**Conclusions:**

The location and morphology of LVT are similar in T2DM and non-diabetic patients. A higher risk of MACCE was found among patients with diabetes.

## 1. Background

An echo-dense mass known as left ventricular thrombus (LVT) that has borders distinct from the endocardium and is usually found close to a segment that is contracting abnormally [1, 2]. LVT is found in 10%–33% of acute myocardial infarction (AMI) patients [2]. Previous research suggested that patients with LVT had poor clinical outcomes and were at an elevated risk of developing major adverse cardiac and cerebrovascular events (MACCE) [3, 4, 5, 6, 7]. Studies linking LVT to heart failure have also been published, indicating that LVT is a sign of left ventricular dysfunction [2, 3, 7, 8].

Like coronary artery disease and heart failure [9], diabetes and LVT often co-exist. According to previous research, the prevalence of diabetes mellitus (DM) in LVT patients ranged from 23.9% [10] to 46.0% [4]. Diabetes is one of the independent risk factors for the emergence of heart failure [9, 11, 12]. Concentric left ventricular remodeling is typically seen in people with type 2 diabetes mellitus (T2DM) and is linked to poor cardiovascular prognosis [13]. However, no research has been done on how DM affects LVT. There is yet no information on the differences between LVT patients with T2DM and those without diabetes.

In particular, the changes in LVT features, long-term clinical outcomes, and differential effects of direct oral anticoagulants (DOAC) between individuals with T2DM and without diabetes were the focus of this study’s investigation into the impact of T2DM on LVT.

## 2. Methods

### 2.1 Study Sample

Patients diagnosed with LVT between 2009 and 2021 in Fuwai hospital according to International Classification of Diseases (ICD) codes were retrospectively included. Fuwai hospital is a national tertiary A-level hospital specializing in cardiovascular diseases and is the world’s largest cardiovascular science center [14]. Participants were split into the DM group and the non-diabetic group. The following were the DM diagnostic criteria [15]: fasting plasma glucose ≥7.0 mmol/L; the 2-h plasma glucose of the oral glucose tolerance test ≥11.1 mmol/L; those with hemoglobin A1c (HbA1c) ≥6.5% at baseline; those with classic symptoms of hyperglycemia or hyperglycemic crisis, a random plasma glucose ≥11.1 mmol/L; or current use of hypoglycemic drugs or insulin. The exclusion criteria were (1) without enough imaging evidence; (2) in-hospital death; (3) atrial thrombus; (4) right ventricular thrombus; (5) left ventricle reconstruction or LVT removal; (6) heart transplantation; or (7) lost follow up.

This study was approved by the Ethics Committee of Fuwai Hospital and was conducted according to the Declaration of Helsinki. Because there was little patient risk, written consent was waived. Verbal consent was gained during the telephone interview.

### 2.2 Clinical Data Collection

The hospital’s electronic medical records system was used to collect medical records, including medical history, test results, and the findings of an echocardiogram. The estimated glomerular filtration rate (eGFR) was determined using the creatine equation from the Chronic Kidney Disease Epidemiology Collaboration (CKD-EPI) [16]. Laboratory test results were at baseline. Comorbid conditions were identified based on ICD codes.

### 2.3 Assessment of LVT

This retrospective cohort evaluated LVT by transthoracic echocardiography, contrast-enhanced CT, or cardiac magnetic resonance imaging (MRI). Participants received echocardiography at the time of admission. LVT was diagnosed on echocardiography using established criteria [17, 18, 19]: a mass within the left ventricular cavity with margins distinct from ventricular endocardium and distinguishable from papillary muscles, chordae, trabeculations, or technical artifacts. To distinguish from tumor, LVT was defined as a left ventricular mass with tissue characteristics consistent with avascular tissue, identifiable as a low-signal-intensity mass surrounded by high-signal-intensity structures such as cavity blood and/or surrounding myocardium. The physicians determined whether to perform MRI or contrast-enhanced CT. The imaging data were independently assessed by two skilled cardiologists. They estimated the location, shape, density, activity, and quantity. A round LVT was defined as a thrombus with a protruding element. The rest were described as mural LVT. Data including left ventricular end-diastolic dimension, left ventricular ejection fraction (LVEF), and wall motion were also collected.

### 2.4 Outcomes and Follow-Up

The primary outcome were MACCE, the composite of cardiovascular death, ischemic stroke, and AMI. The secondary endpoints were all-cause death and cardiovascular death. To find out about negative outcomes, phone calls were made to each patient. Information was obtained from accessible medical records and redacted at the time of the patient’s most recent outpatient visit or hospital discharge when patients could not be reached. Time zero for the statistical analyses was the date of discharge from the hospital.

### 2.5 Statistical Analysis

Continuous variables are represented as median (interquartile range, IQR) or means (standard deviations, SD). Mann–Whitney’s U-test or Student’s *t*-test was implemented to compare continuous variables. Categorical variables were expressed as number (percentage) and compared using the Chi-square Test or Fisher Exact Probability Test as appropriate. Survival analysis was performed using the cumulative incidence functions (CIF) method. The Gray’s test was used to compare the two groups. The multivariable analysis was conducted to assess hazard ratio and control potential confounding. Factors were selected because of the notable variations in baseline features between groups or their probable relationship to prognosis. A two-tailed *p *≤ 0.05 was considered statistically significant. All analyses were performed with R 4.2.1 (R Core Team, Vienna, Austria).

We employed propensity score matching (PSM) to equalize the baseline features. Multivariable logistic regression model was used to calculate the propensity score. The **Supplementary Methods** report the model in detail. To determine how well PSM reduced the baseline discrepancy, the standardized mean difference (SMD) was used. An SMD ≤0.1 suggested a useful PSM. Inverse probability of treatment weighting (IPTW)was also utilized.

## 3. Results

### 3.1 Baseline Characteristics

**Fig. 1.**
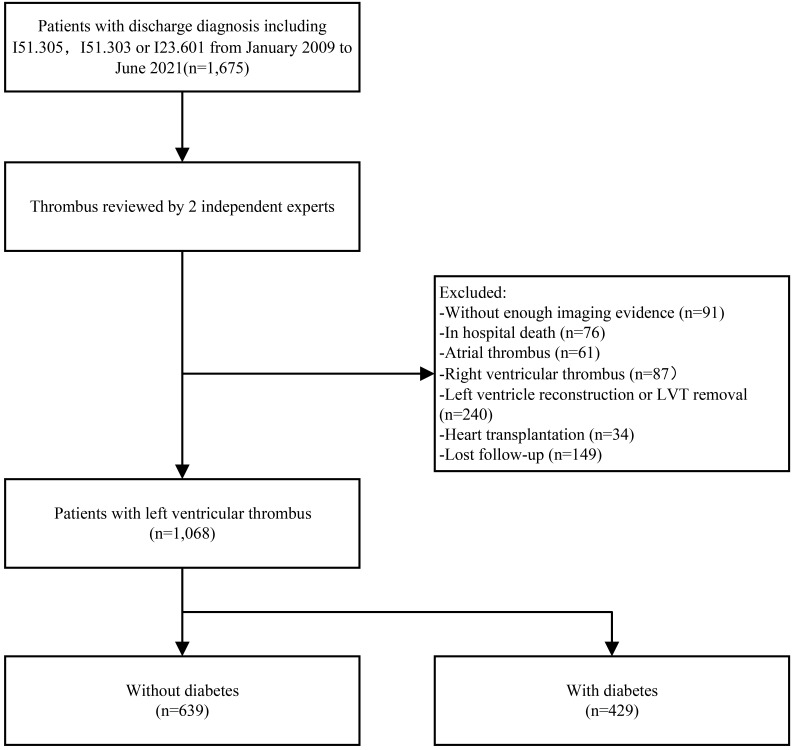
**Study flow chart**. LVT, left ventricular thrombus.

This study comprised 1068 participants, of which 429 had diabetes, and 639 did not. Fig. 1 depicts the flowchart of the study’s selection process. All 429 diabetic patients were T2DM. The baseline features of the T2DM group and the non-diabetic group differ in that (a) patients with LVT combined with T2DM were significantly older (56 vs. 51, *p *< 0.001) and (b) a substantially higher proportion were female (80.4% vs. 85.3%, *p* = 0.044). Particularly higher ratios of comorbidities were also observed in the T2DM group, including hypertension (54.5% vs. 42.4%, *p *< 0.001), eGFR <60 mL/min/1.73 $m^{2}$ (21.9% vs. 10.0%, *p *< 0.001), prior stroke (19.8% vs. 12.7%, *p* = 0.002), prior MI (58.0% vs. 49.9%, *p* = 0.011), atrial fibrillation (12.8% vs. 5.9%, *p *< 0.001), and coronary artery disease (80.7% vs. 72.5%, *p* = 0.003). The antithrombotic treatment at discharge did not differ between the two groups.

Notably, the LVEF was significantly lower in the T2DM group than in those without diabetes (36% vs. 39%, *p* = 0.031). The location, morphology, and size of LVT were similar in the two groups. Table 1 provides specifics of the study participants’ baseline characteristics. Clinical demographics of follow-up cases and loss to follow-up cases were shown in **Supplementary Table 1**. Patients lost to follow-up had a higher proportion of prior MI (65.6% vs. 53.2%, *p* = 0.011), a lower ratio of STEMI (12.8% vs. 21.3%, *p* = 0.035), and global hypokinesis (17.6% vs. 26.1%, *p* = 0.049).

**Table 1.** **Baseline characteristics according to type 2 diabetes mellitus**.

		Without T2DM	With T2DM	*p* value	SMD
n		639	429		
Demographics					
	Age/years	50.71 (15.35)	56.34 (13.47)	<0.001	0.39
	Male	545 (85.3)	345 (80.4)	0.044	0.129
	Body mass index/kg/$m^{2}$	24.82 [22.30, 27.37]	25.10 [23.24, 27.68]	0.083	0.116
Past medical history					
	Hypertension	271 (42.4)	234 (54.5)	<0.001	0.245
	eGFR <60 mL/min/1.73 $m^{2}$	64 (10.0)	94 (21.9)	<0.001	0.329
	Peripheral artery disease	40 (6.3)	39 (9.1)	0.107	0.107
	Prior stroke	81 (12.7)	85 (19.8)	0.002	0.194
	Prior MI	319 (49.9)	249 (58.0)	0.011	0.163
	Prior CABG	11 (1.7)	12 (2.8)	0.331	0.072
	Prior PCI	87 (13.6)	78 (18.2)	0.053	0.125
	Prior cerebral hemorrhage	4 (0.6)	3 (0.7)	1	0.009
	Atrial fibrillation	38 (5.9)	55 (12.8)	<0.001	0.237
Underlying diseases					
	Coronary artery disease	463 (72.5)	346 (80.7)	0.003	0.194
	STEMI	148 (23.2)	79 (18.4)	0.075	0.117
	NSTEMI	23 (3.6)	22 (5.1)	0.287	0.075
	Dilated cardiomyopathy	107 (16.7)	71 (16.6)	1	0.005
	Hypertrophic cardiomyopathy	18 (2.8)	8 (1.9)	0.431	0.063
	ARVD with associated LV impairment	6 (0.9)	1 (0.2)	0.31	0.093
	Perinatal cardiomyopathy	13 (2.0)	2 (0.5)	0.062	0.141
	Restrictive cardiomyopathy	3 (0.5)	2 (0.5)	1	<0.001
	Alcoholic cardiomyopathy	11 (1.7)	3 (0.7)	0.244	0.094
	Myocarditis	5 (0.8)	2 (0.5)	0.809	0.04
	NVM	16 (2.5)	10 (2.3)	1	0.011
Medications					
	Aspirin	359 (56.2)	244 (56.9)	0.872	0.014
	Clopidogrel	296 (46.3)	208 (48.5)	0.528	0.043
	Ticagrelor	24 (3.8)	16 (3.7)	1	0.001
	DAPT	263 (41.2)	174 (40.6)	0.895	0.012
	VKA	228 (35.7)	148 (34.5)	0.741	0.025
	Rivaroxaban	137 (21.4)	100 (23.3)	0.518	0.045
	Dabigatran	16 (2.5)	8 (1.9)	0.631	0.044
	DOAC	154 (24.1)	108 (25.2)	0.743	0.025
	Antiplatelet therapy only	234 (36.6)	160 (37.3)	0.873	0.014
	Anticoagulation only	200 (31.3)	122 (28.4)	0.352	0.063
	Anticoagulation status			0.731	0.178
	Dabigatran 110 mg BID	15 (2.3)	8 (1.9)		
	Dabigatran 150 mg BID	1 (0.2)	0 (0.0)		
	Rivaroxaban 2.5 mg QD	4 (0.6)	6 (1.4)		
	Rivaroxaban 5 mg QD	4 (0.6)	3 (0.7)		
	Rivaroxaban 5 mg BID	0 (0.0)	2 (0.5)		
	Rivaroxaban 10 mg QD	9 (1.4)	10 (2.3)		
	Rivaroxaban 10 mg BID	1 (0.2)	2 (0.5)		
	Rivaroxaban 15 mg QD	41 (6.4)	28 (6.5)		
	Rivaroxaban 15 mg BID	16 (2.5)	11 (2.6)		
	Rivaroxaban 20 mg QD	62 (9.7)	38 (8.9)		
	Aspirin with anticoagulant	54 (8.5)	43 (10.0)	0.442	0.054
	Clopidogrel with anticoagulant	52 (8.1)	43 (10.0)	0.341	0.066
	Ticagrelor with anticoagulant	1 (0.2)	0 (0.0)	1	0.056
	Anticoagulant with dual antiplatelet therapy	75 (11.7)	48 (11.2)	0.859	0.017
Imaging morphology of LVT					
	LVEDD	58.00 [52.00, 66.00]	58.00 [54.00, 66.00]	0.255	0.041
	LVEF	39.00 [29.00, 47.00]	36.00 [28.00, 45.00]	0.031	0.137
	LVEF ≤40%	376 (58.8)	278 (64.8)	0.058	0.123
	Global hypokinesis	168 (26.3)	111 (25.9)	0.935	0.009
	Hypokinesis	266 (41.6)	194 (45.2)	0.271	0.073
	Akinesis	380 (59.5)	258 (60.1)	0.876	0.014
	Apical LVT	586 (91.7)	381 (88.8)	0.139	0.098
	Round LVT	386 (60.4)	266 (62.0)	0.645	0.033
	Mobile LVT	50 (7.8)	39 (9.1)	0.535	0.046
	Multiple LVT	69 (10.8)	51 (11.9)	0.65	0.034
	Calcified LVT	109 (17.1)	86 (20.0)	0.247	0.077
	LVT largest diameter/mm	23.00 [17.00, 30.00]	24.00 [16.00, 33.00]	0.123	0.101
	LVT area/${\mathrm{Mm}}^{2}$	2.88 [1.65, 4.48]	3.15 [1.62, 5.20]	0.189	0.108
	Left ventricular aneurysm	318 (49.8)	216 (50.3)	0.901	0.012

Data are n/N (%), median (IQR) or mean (SD). T2DM, type 2 diabetes mellitus; SMD, standard mean difference; eGFR, estimated glomerular filtration rate; MI, myocardial infarction; CABG, coronary artery bypass grafting; PCI, percutaneous coronary intervention; STEMI, ST-segment elevation myocardial infarction; NSTEMI, non-ST-segment elevation myocardial infarction; ARVD, arrhythmogenic right ventricular dysplasia; NVM, noncompaction of the ventricular myocardium; DAPT, dual antiplatelet therapy; VKA, vitamin-K antagonists; DOAC, direct oral anticoagulants; LVEDD, left ventricular end diastolic dimension; LVEF, left ventricular ejection fraction; LVT, left ventricular thrombus.

PSM analysis matched 382 pairs of patients, whereas IPTW analysis resulted in 1058.69 participants (636.52 without T2DM, 422.17 with T2DM). Baseline features were balanced after the matching and weighting analysis (SMD <0.100; *p *< 0.05) (**Supplementary Tables 2,3** and **Supplementary Fig. 1**).

### 3.2 Primary and Secondary Outcomes

**Fig. 2.**
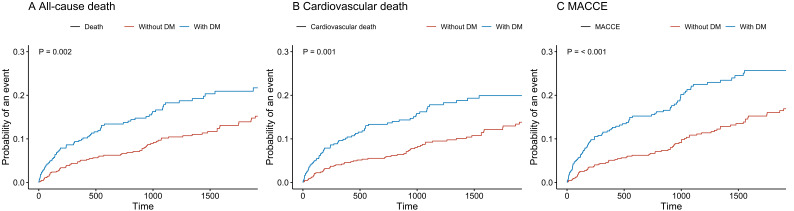
**Survival curves according to diabetes mellitus**. (A) All-cause death; (B) Cardiovascular death; (C) MACCE. DM, type 2 diabetes mellitus; MACCE, major adverse cardiac and cerebrovascular events.

Follow-up data were available for 91.1% (1526) of the study participants. The median follow-up time was 3.8 (IQR = 1.9–6.6) years. Of 182 all-cause deaths, 164 were cardiovascular deaths, and 203 MACCE occurred. The time-to-event curves to estimate the event rate are shown in Fig. 2. The cumulative risk of all-cause death (20.3% vs. 14.9%; hazard ratio [HR] 1.58, 95% confidence interval [CI] 1.18–2.11, *p* = 0.002), cardiovascular death (18.9% vs. 13.0%; HR 1.66, 95% CI 1.22–2.25, *p* = 0.001) and MACCE (23.8% vs. 15.8%; HR 1.75, 95% CI 1.33–2.30, *p *< 0.001) was significantly higher in the T2DM group. The result of multivariable CIF suggested a higher risk of MACCE (HR 1.43, 95% CI 1.06–1.92, *p* = 0.018) among patients with diabetes (Table 2). A *post hoc* collinearity analysis of the variables included in the multivariable regression model is provided in the **Supplementary Table 4** (variance inflation factor) and **SupplementaryTable 5** (correlation matrix). The maximum variance inflation factor and correlation coefficient were 1.39 (<10) and 0.38 (<0.4), indicating that the included variables were not significantly correlated.

**Table 2.** **Outcomes in the whole sample**.

	Overall (*n* = 1068)	Without T2DM (*n* = 639)	With T2DM (*n *= 429)	Univariable analysis		Multivariable analysis		PSM analysis		IPTW analysis	
				Hazard ratio (95% CI)	*p* values	Hazard ratio (95% CI)	*p* values	Hazard ratio (95% CI)	*p* values	Hazard ratio (95% CI)	*p* values
All-cause death	182 (17.0%)	95 (14.9%)	87 (20.3%)	1.58 (1.18–2.11)	0.002	1.28 (0.93–1.76)	0.13	1.15 (0.82–1.62)	0.4	1.16 (0.85–1.60)	0.352
Cardiovascular death	164 (15.4%)	83 (13.0%)	81 (18.9%)	1.66 (1.22–2.25)	0.001	1.39 (0.99–1.93)	0.055	1.26 (0.87–1.81)	0.2	1.22 (0.87–1.71)	0.243
MACCE	203 (19.0%)	101 (15.8%)	102 (23.8%)	1.75 (1.33–2.30)	<0.001	1.43 (1.06–1.92)	0.018	1.27 (0.91–1.76)	0.15	1.29 (0.95–1.74)	0.101
Stroke	32 (3.0%)	14 (2.2%)	18 (4.2%)	2.10 (1.05–4.18)	0.035	1.79 (0.91–3.51)	0.090	1.13 (0.50–2.54)	0.8	1.76 (0.84–3.70)	0.138
Acute MI	18 (1.7%)	7 (1.1%)	11 (2.6%)	2.55 (1.01–6.45)	0.048	2.02 (0.78–5.24)	0.15	3.10 (0.63–15.2)	0.2	1.56 (0.55–4.48)	0.406

Values are n (%). The multivariable hazard ratio is adjusted for age, gender, eGFR <60 mL/min/1.73 $m^{2}$, body mass index, ejection fraction ≤40%, hypertension, prior myocardial infarction, prior stroke, and atrial fibrillation. T2DM, type 2 diabetes mellitus; MACCE, major adverse cardiac and cerebrovascular events; MI, myocardial infarction; CI, confidence interval.

After multivariable adjustment, T2DM (HR 1.43, 95% CI 1.06–1.92, *p* = 0.018), eGFR <60 mL/min/1.73 $m^{2}$ (HR 2.42, 95% CI 1.68–3.47, *p *< 0.001), prior stroke (HR 1.75, 95% CI 1.25–2.46, *p* = 0.001), and LVEF ≤40% (HR 2.46, 95% CI 1.76–3.43, *p *< 0.001) were associated with an increased risk of MACCE (**Supplementary Fig. 2**).

To assess the robustness of the results, a univariable model was also performed in the PSM and IPTW analyses. The findings demonstrated that T2DM was not linked to all-cause mortality (PSM: HR 1.15, 95% CI 0.82–1.62, *p* = 0.4; IPTW: HR 1.16, 95% CI 0.85–1.60, *p* = 0.352), cardiovascular death (PSM: HR 1.26, 95% CI 0.87–1.81, *p* = 0.2; IPTW: HR 1.22, 95% CI 0.87–1.71, *p* = 0.243) and MACCE (PSM: HR 1.27, 95% CI 0.91–1.76, *p* = 0.15; IPTW: HR 1.29, 95% CI 0.95–1.74, *p* = 0.101).

We also compared these events, including acute MI and stroke; T2DM was associated with increased risk of acute MI (HR 2.55, 95% CI 1.01–6.45, *p* = 0.048) and stroke (HR 2.10, 95% CI 1.05–4.18, *p* = 0.035) only in univariate analysis.

### 3.3 Differential Effects of Direct Oral Anticoagulants

**Fig. 3.**
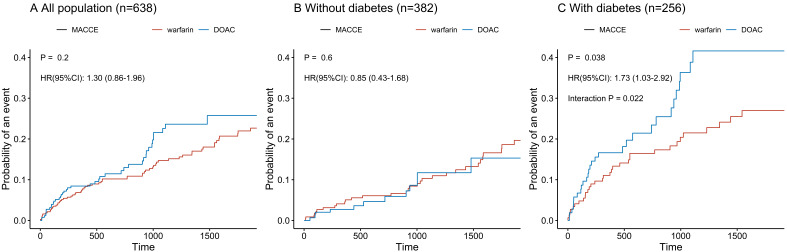
**Comparison of risk of major adverse cardiac and cerebrovascular events according to DOAC vs. warfarin treatment according to type 2 diabetes mellitus status in patients receiving anticoagulation**. (A) All sample (*n* = 638). (B) Without diabetes (*n* = 382). (C) With diabetes (*n* = 256). DOAC, direct oral anticoagulants; HR, hazard ratio; CI, confidence interval; MACCE, major adverse cardiac and cerebrovascular events. The presented hazard ratio is multivariable adjusted for age, gender, eGFR <60 mL/min/1.73 $m^{2}$, body mass index, ejection fraction ≤40%, hypertension, prior myocardial infarction, prior stroke, and atrial fibrillation.

Among the group with LVT and anticoagulation at discharge (*n* = 638), the incidence of MACCE did not differ between those receiving DOAC treatment and those receiving warfarin treatment (HR 1.30, 95% CI 0.86–1.96, *p* = 0.2) (Fig. 3A). Among the non-diabetic LVT participants with anticoagulation (*n* = 382), the incidence of MACCE was also similar in the two groups (HR 0.85, 95% CI 0.43–1.68, *p* = 0.6) (Fig. 3B). However, in the diabetic LVT population with anticoagulation (*n* = 256), DOAC treatment was associated with a significantly higher risk of MACCE than was warfarin treatment (HR 1.73, 95% CI 1.03–2.92, *p* = 0.038) (Fig. 3C). There was a significant interaction between the use of DOAC and the presence of diabetes for the risk of MACCE (interaction *p* = 0.022).

Survival curves of all three endpoints grouped according to DOAC and warfarin are shown in** Supplementary Fig. 3** (in LVT patients receiving anticoagulation), **Supplementary Fig. 4** (in non-diabetic LVT patients receiving anticoagulation), and **Supplementary Fig. 5** (in diabetic LVT patients receiving anticoagulation). **SupplementaryTable 6** shows the multivariable analysis for the relationship between DOAC and adverse outcomes in LVT patients receiving anticoagulation. DOAC tends to increase MACCE in people with diabetes (Interaction P of MACCE = 0.022).

## 4. Discussion

As far as we know, no previous article has compared the variations between LVT both with and without T2DM. This is the first study to assess the differences between T2DM and non-diabetic patients in a large cohort of LVT patients. Additionally, this is the first investigation into the relationship between DOAC and diabetes in LVT patients. In this cohort analysis of 1068 LVT patients, we established (1) the location and morphology of LVT are similar in T2DM and non-diabetic patients; (2) people with T2DM have a worse cardiovascular prognosis; (3) DOAC treatment may increase the risk of MACCE in patients with LVT and T2DM.

Patients in the T2DM group tended to be much older, have lower LVEF, more hypertension, have a history of stroke, have atrial fibrillation, and have worse eGFR. Additionally, the T2DM group had greater proportions of prior MI and were paired with more coronary artery disease, which suggests a higher atherosclerotic burden. This was not surprising because metabolic syndrome includes T2DM [20], as well as complications in other systems [21]. Similar morphology was found in the T2DM group using imaging, including ultrasound. Survival curves, univariable, and multivariable analyses showed that diabetes increased MACCE in patients with LVT. However, the difference in risk between the two groups after matching and weighting was not statistically significant. This may be due to the insufficient sample size, although our cohort is the largest cohort of LVT to date. More than half of the patients in our LVT cohort had an LVEF <40%. The main cause of death was heart failure.

No prior studies compare the differences in LVT patients with T2DM and without diabetes. The coexistence of heart failure and T2DM is common and strongly impacts clinical management and prognosis. In individuals with heart failure and reduced or preserved ejection fraction, T2DM is linked to a worse clinical state and increased all-cause and cardiovascular mortality than in people without T2DM [9, 22, 23]. T2DM and heart failure patients in the CHARM trial had higher mortality rates across all subtypes of cardiovascular death [24]. According to the PARADIGM-HF trial, those with heart failure and diabetes were more likely to die from cardiovascular and other causes than people without diabetes [25].

It is important to note that various alterations can cause cardiovascular damage including those that affect the metabolism, the kidneys, the myocardium, the endothelium, and the inflammatory systems [23]. According to a widely accepted model, the interaction of three factors—stasis caused by diminished ventricular function, endocardial damage, and hypercoagulability—leads to the etiology of LVT [26]. The diabetic prothrombotic condition is caused by a number of processes, such as platelet hyperactivity, coagulative activation, and endothelial dysfunction [27, 28]. First, hyperactivity of platelets, or enhanced responsiveness of platelets, has been proposed as a key factor in the development of cardiovascular problems in diabetes. The finding of elevated levels of thromboxane B2 in the urine of T2DM patients suggests platelet hyperactivity [29, 30]. In T2DM, there is a decrease in the expression of the receptor for the negative platelet regulator prostacyclin, which improves platelet responsiveness [31]. Second, patients with T2DM are more likely to have hypercoagulable states due to altered plasma levels of coagulation factors [32]. At the same time, T2DM results in less fibrinolysis, the process by which clots dissolve [33]. The over-coagulative status could be caused by DM in patients with the lowest thrombotic risk score and atrial fibrillation [34] and those with acute coronary syndrome [35, 36]. In both cases, the over-thrombosis could be caused by endothelial dysfunction, increased platelet aggregation, and over-activation of the inflammatory cascade and prothrombotic pathways. Diabetes play an important role on the alteration of microbiota, and the microbiota thrombus colonization, then influencing the athero-thrombosis and leading to worse clinical outcomes [37]. However, no difference in LVT size was observed between our two groups of patients. Third, the increase in platelet adhesion and clot formation is the overall result of T2DM-dependent endothelial cell injury. The increased thrombotic risk for T2DM patients is a result of the endothelial cell-dependent modulation of platelets and fibrinolysis [38].

DOAC treatment has been widely used in the whole population with LVT [8, 39, 40, 41]. Moreover, our study suggests that DOAC can increase MACCE in patients with LVT and diabetes. Given the relatively small number of patients with LVT, no one has compared DOAC treatment with vitamin-K antagonists (VKA) in a diabetic subgroup and investigated the interaction of them. Previous studies focused on the LVT patients’ anticoagulation with DOAC and VKA [42, 43]. Rivaroxaban was shown to be comparable to warfarin in the NO-LVT Trial, and to have a faster rate of thrombus clearance, in patients from Egypt and Bulgaria [44]. According to an Israeli study, there is a 20% non-inferiority margin between apixaban and warfarin for treating patients with LVT after an acute MI [45]. However, the sample size of these two RCTs was small, the follow-up time was short, and the primary endpoint was not a hard endpoint. A newly published meta-analysis comprising 21 studies (*n *= 3172, 3 RCTs, 18 observational studies) found that compared with VKA, DOAC dramatically reduce the risk of bleeding events and stroke in LVT patients. Still, mortality was comparable in the two groups [42].

In patients with atrial fibrillation and T2DM, non-vitamin K antagonist oral anticoagulants produced reduced diabetes complications and mortality risk than did warfarin [46, 47]. Our study suggests that the efficacy of DOAC is different in patients with LVT and T2DM than in patients with atrial fibrillation and T2DM. Some potential mechanisms could explain why DOAC is inferior to warfarin in T2DM patients with LVT. First, unlike the treatment of atrial fibrillation, DOAC treatment has no specific dose recommendation in the treatment of LVT, which was confirmed in our study. In patients with atrial fibrillation, non-recommended DOAC doses were associated with an increased risk of death [48, 49]. Second, confounding problems with different DOAC may lead to reduced efficacy. No randomized clinical trials have compared different DOAC head-to-head. In a retrospective cohort analysis, Ray and associates compared the effectiveness of rivaroxaban with that of apixaban in treating atrial fibrillation [50]. They concluded that patients who received rivaroxaban had 2.7 additional adverse outcomes (95% CI 1.9–3.5) and 21.1 other nonfatal bleeding events (95% CI 20.0–22.3) over 1000 patient-years of treatment, than did those who received apixaban. Correspondingly, the application of rivaroxaban in our study was dominant in DOAC treatment. Third, like the INVICTUS trial [51], the lower MACCE in the VKA group is speculated to be related to the monthly INR monitoring and frequent contact and interaction with doctors to get better whole-course care. Future RCT studies, especially in diabetic samples, are needed. Given the small sample size in certain subgroups, our result needs to be interpreted with caution.

## 5. Limitation

This study has several limitations. First, it is important to acknowledge the limitations of an observational cohort study conducted in a single center. The key limitations relate to the retrospective nature of our research, which was not a head-to-head comparison of anticoagulants. This may limit the potential generalizability to other populations. Therefore, our findings should be considered hypothesis-generating. Second, despite efforts to correct confounding variables, there are likely to be residual confounders that we have been unable to fix, such as the anticoagulation adherence and duration, treatment switching between DOAC and VKA, and time in therapeutic range during the follow-up. Third, major bleeding events and the resolution of LVT between groups were not analyzed. Finally, we enrolled patients over ten years. Hence, the cohort of patients enrolled in the later part of the study will have shorter follow-ups and less time to report events.

## 6. Conclusions

This is the first study to investigate differences in LVT characteristics, clinical outcomes, and differential effects of DOAC treatment among patients with T2DM and without diabetes. The location and morphology of LVT are similar between diabetic and non-diabetic patients. A higher risk of MACCE was found among patients with type 2 diabetes. The off-label use of DOAC, the main rivaroxaban, is popular in diabetic patients. However, DOAC may increase the risk of MACCE in patients with LVT and type 2 diabetes.

## Data Availability

Due to privacy and ethical concerns, the datasets used in the current work cannot be made publicly available. However, the corresponding author can provide them upon reasonable request.

## Abbreviations

LVT, left ventricular thrombus; MACCE, major adverse cardiac and cerebrovascular events; DM, diabetes mellitus; T2DM, type 2 diabetes mellitus; DOAC, direct oral anticoagulants; MI, myocardial infarction; SE, systemic embolism; LVEF, left ventricular ejection fraction; VKA, vitamin-K antagonists.

## Supplementary Material

Supplementary material associated with this article can be found, in the online version, at https://doi.org/10.31083/j.rcm2403065.
