# Supplementary material for: Impacts of Diabetes Mellitus on Cardiovascular Outcomes and Differential Effects of Direct Oral Anticoagulants in Patients with Left Ventricular Thrombus
Source: Rev Cardiovasc Med. 2023 Feb 22;24(3):65. doi: 10.31083/j.rcm2403065 (PMC11264001; doi:10.31083/j.rcm2403065)
Supplement: Supplementary file 1 [file 2153-8174-24-3-065-s1.docx]

**SUPPLEMENTAL MATERIALS**

**1.Supplemental Methods**

The variables included in the model estimating propensity score are: age, gender, body mass index, hypertension, diabetes mellitus, eGFR<60ml/min/1.73m^2^, peripheral artery disease, prior stroke, prior MI, prior CABG, prior PCI, prior cerebral hemorrhage, atrial fibrillation, coronary artery disease, STEMI, NSTEMI, dilated cardiomyopathy, hypertrophic cardiomyopathy, ARVD with associated LV impairment, perinatal cardiomyopathy, restrictive cardiomyopathy, alcoholic cardiomyopathy, myocarditis, aspirin, clopidogrel, ticagrelor, DAPT, VKA, rivaroxaban, dabigatran, DOAC, antiplatelet therapy only, anticoagulation only, aspirin with anticoagulant, clopidogrel with anticoagulant, ticagrelor with anticoagulant, dual antiplatelet therapy with anticoagulant, NVM, LVEDD, LVEF, LVEF<=40%, global hypokinesis, hypokinesis, akinesis, apical LVT, round LVT, mobile LVT, multiple LVT, calcified LVT, LVT largest diameter, LVT area, left ventricular aneurysm.

**2.Supplemental Figures**


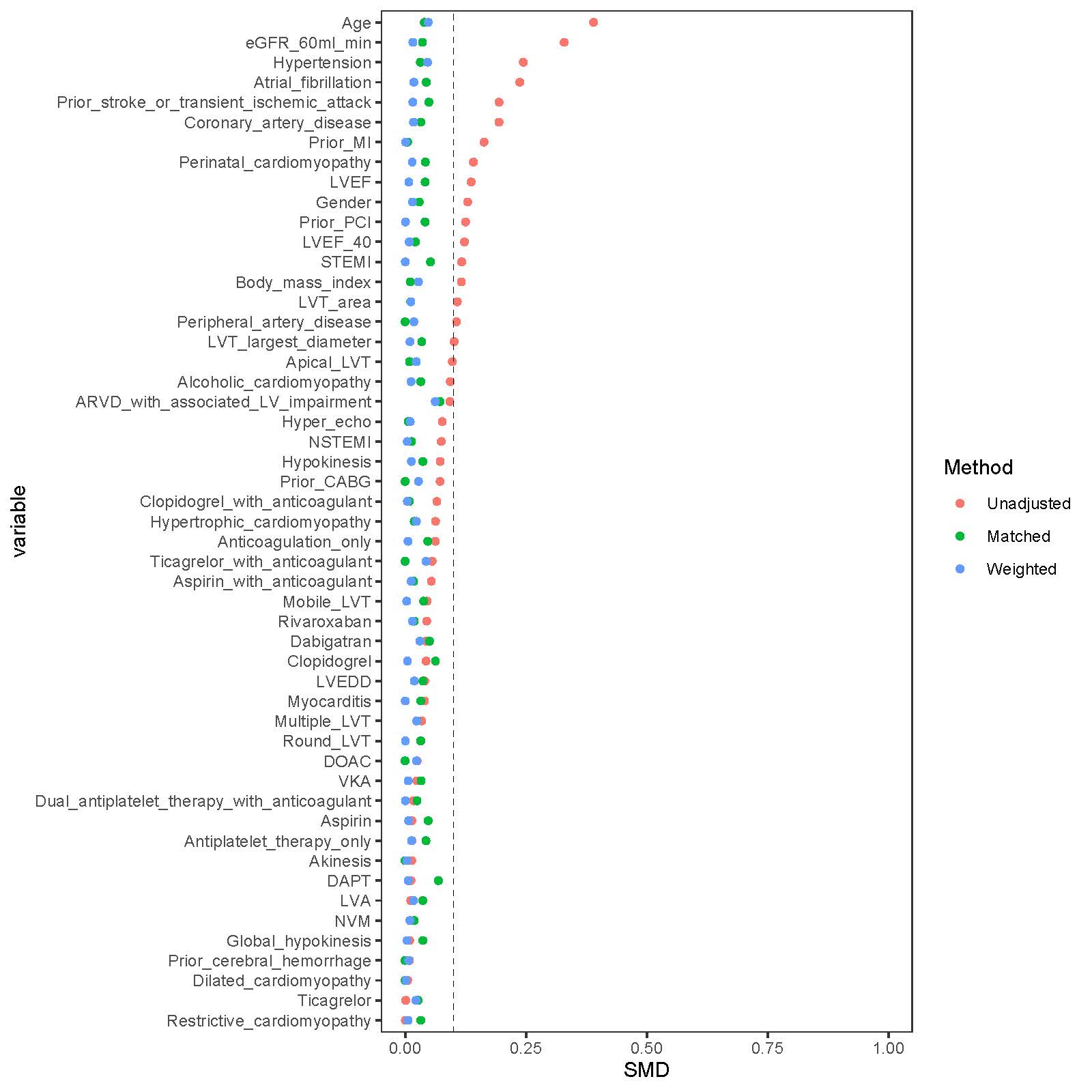


**Supplemental Figure 1 Standardized mean difference (SMD) between the 2 groups before and after propensity score matching and propensity score weighting.**

SMD=standard mean difference. eGFR=estimated glomerular filtration rate. MI=myocardial infarction. CABG=coronary artery bypass grafting. PCI=percutaneous coronary intervention. STEMI=ST-segment elevation myocardial infarction. NSTEMI=non-ST-segment elevation myocardial infarction. ARVD=arrhythmogenic right ventricular dysplasia. NVM=noncompaction of the ventricular myocardium. DAPT=dual antiplatelet therapy. VKA=vitamin-K antagonists. DOAC=direct oral anticoagulants. LVEDD=left ventricular end diastolic dimension. LVEF=left ventricular ejection fraction. LVT=left ventricular thrombus.


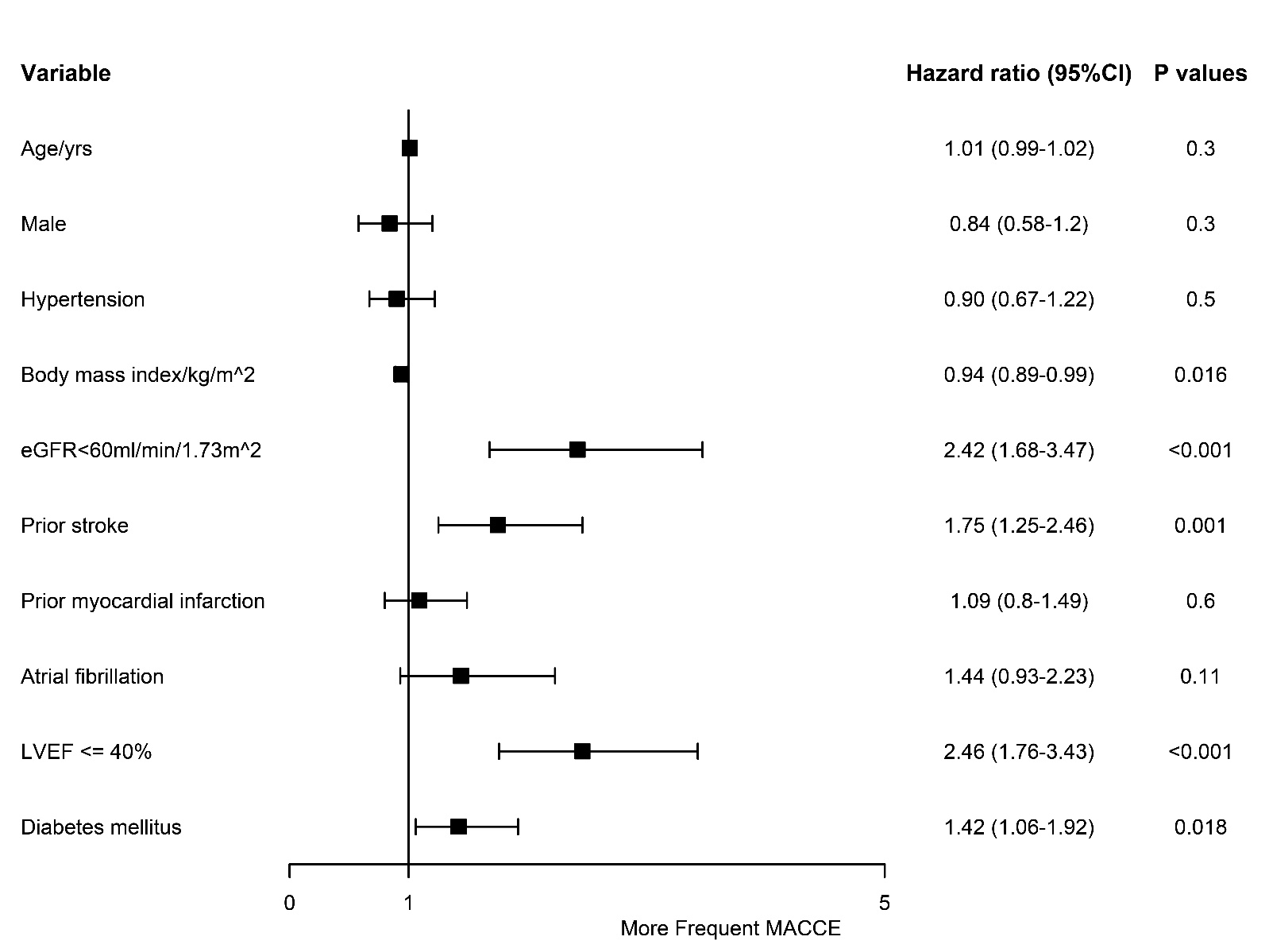


**Supplemental Figure 2 Association between baseline characters and the risk of major adverse cardiac and cerebrovascular events by multivariable analysis.**

CI=confidence interval. eGFR=estimated glomerular filtration rate. LVEF=left ventricular ejection fraction.

**Supplemental Figure 3 Survival curves for adverse outcomes in LVT patients receiving anticoagulation.**


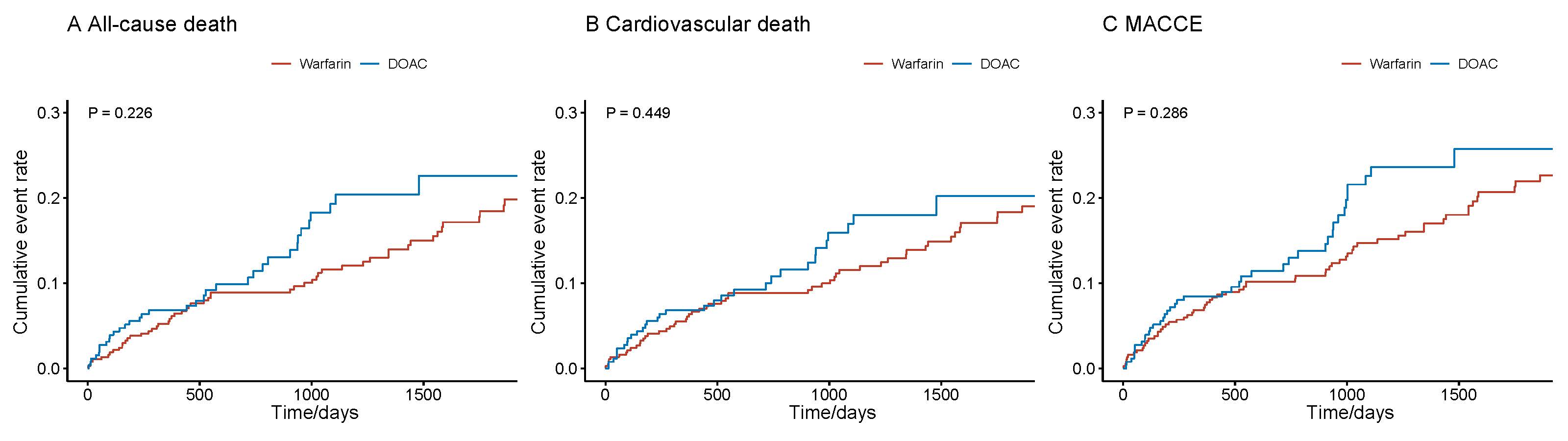
LVT=left ventricular thrombus. DOAC=direct oral anticoagulants. MACCE=major adverse cardiac and cerebrovascular events.

**Supplemental Figure 4 Survival curves for adverse outcomes in non-diabetic LVT patients receiving anticoagulation.**


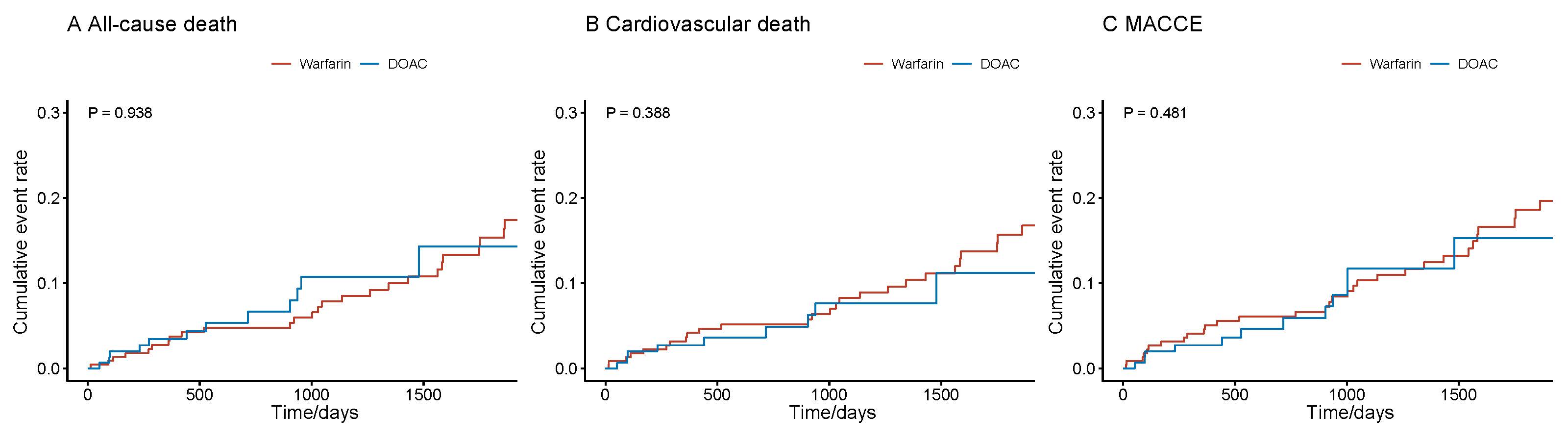
LVT=left ventricular thrombus. DOAC=direct oral anticoagulants. MACCE=major adverse cardiac and cerebrovascular events.

**Supplemental Figure 5 Survival curves for adverse outcomes in diabetic LVT patients receiving anticoagulation.**


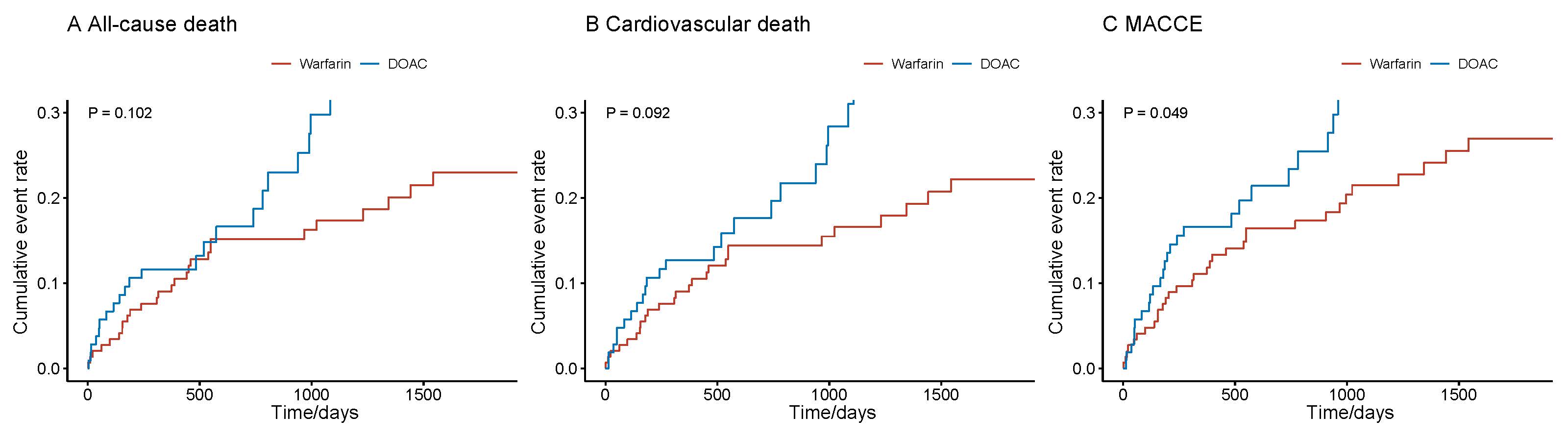
LVT=left ventricular thrombus. DOAC=direct oral anticoagulants. MACCE=major adverse cardiac and cerebrovascular events.

**3.Supplemental Tables**

**Supplemental Table 1 Baseline features** **of follow-up and lost to follow-up cases.**

|  | Overall | Lost to follow-up cases | Follow-up cases | P value | SMD |
| --- | --- | --- | --- | --- | --- |
| n | 1,193 | 125 | 1,068 |  |  |
| **Demographic** |  |  |  |  |  |
| Age/years | 53.21 (14.72) | 55.26 (13.19) | 52.97 (14.88) | 0.099 | 0.163 |
| Male | 999 (83.7) | 109 (87.2) | 890 (83.3) | 0.327 | 0.109 |
| Body mass index/kg/m^2^ | 25.00 [22.60, 27.47] | 24.61 [22.49, 27.46] | 25.03 [22.65, 27.47] | 0.669 | 0.054 |
| **Past medical history** |  |  |  |  |  |
| Hypertension | 561 (47.0) | 56 (44.8) | 505 (47.3) | 0.666 | 0.05 |
| Diabetes mellitus | 490 (41.1) | 61 (48.8) | 429 (40.2) | 0.078 | 0.174 |
| eGFR<60 ml/min/1.73m^2^ | 178 (14.9) | 20 (16.0) | 158 (14.8) | 0.822 | 0.033 |
| Peripheral artery disease | 87 (7.3) | 8 (6.4) | 79 (7.4) | 0.823 | 0.039 |
| Prior stroke | 187 (15.7) | 21 (16.8) | 166 (15.5) | 0.814 | 0.034 |
| Prior MI | 650 (54.5) | 82 (65.6) | 568 (53.2) | 0.011 | 0.255 |
| Prior CABG | 25 (2.1) | 2 (1.6) | 23 (2.2) | 0.937 | 0.041 |
| Prior PCI | 177 (14.8) | 12 (9.6) | 165 (15.4) | 0.108 | 0.177 |
| Prior cerebral hemorrhage | 8 (0.7) | 1 (0.8) | 7 (0.7) | 1 | 0.017 |
| Atrial fibrillation | 102 (8.5) | 9 (7.2) | 93 (8.7) | 0.688 | 0.056 |
| **Underlying disease** |  |  |  |  |  |
| Coronary artery disease | 912 (76.4) | 103 (82.4) | 809 (75.7) | 0.122 | 0.164 |
| STEMI | 243 (20.4) | 16 (12.8) | 227 (21.3) | 0.035 | 0.226 |
| NSTEMI | 49 (4.1) | 4 (3.2) | 45 (4.2) | 0.763 | 0.054 |
| Dilated cardiomyopathy | 195 (16.3) | 17 (13.6) | 178 (16.7) | 0.454 | 0.086 |
| Hypertrophic cardiomyopathy | 28 (2.3) | 2 (1.6) | 26 (2.4) | 0.786 | 0.059 |
| ARVD with associated LV impairment | 7 (0.6) | 0 (0.0) | 7 (0.7) | 0.773 | 0.115 |
| Perinatal cardiomyopathy | 15 (1.3) | 0 (0.0) | 15 (1.4) | 0.363 | 0.169 |
| Restrictive cardiomyopathy | 6 (0.5) | 1 (0.8) | 5 (0.5) | 1 | 0.042 |
| Alcoholic cardiomyopathy | 15 (1.3) | 1 (0.8) | 14 (1.3) | 0.952 | 0.05 |
| Myocarditis | 8 (0.7) | 1 (0.8) | 7 (0.7) | 1 | 0.017 |
| NVM | 27 (2.3) | 1 (0.8) | 26 (2.4) | 0.398 | 0.13 |
| **Medications** |  |  |  |  |  |
| Aspirin | 685 (57.4) | 82 (65.6) | 603 (56.5) | 0.063 | 0.188 |
| Clopidogrel | 561 (47.0) | 57 (45.6) | 504 (47.2) | 0.808 | 0.032 |
| Ticagrelor | 44 (3.7) | 4 (3.2) | 40 (3.7) | 0.956 | 0.03 |
| DAPT | 489 (41.0) | 52 (41.6) | 437 (40.9) | 0.96 | 0.014 |
| VKA | 419 (35.1) | 43 (34.4) | 376 (35.2) | 0.937 | 0.017 |
| Rivaroxaban | 259 (21.7) | 22 (17.6) | 237 (22.2) | 0.288 | 0.115 |
| Dabigatran | 25 (2.1) | 1 (0.8) | 24 (2.2) | 0.46 | 0.118 |
| DOAC | 285 (23.9) | 23 (18.4) | 262 (24.5) | 0.158 | 0.15 |
| Antiplatelet therapy only | 446 (37.4) | 52 (41.6) | 394 (36.9) | 0.351 | 0.097 |
| Anticoagulation only | 349 (29.3) | 27 (21.6) | 322 (30.1) | 0.06 | 0.196 |
| Anticoagulation status |  |  |  | 0.227 | 0.351 |
| Dabigatran 110 mg BID | 24 (2.0) | 1 (0.8) | 23 (2.2) |  |  |
| Dabigatran 150 mg BID | 1 (0.1) | 0 (0.0) | 1 (0.1) |  |  |
| Rivaroxaban 2.5 mg QD | 15 (1.3) | 5 (4.0) | 10 (0.9) |  |  |
| Rivaroxaban 5 mg QD | 7 (0.6) | 0 (0.0) | 7 (0.7) |  |  |
| Rivaroxaban 5 mg BID | 2 (0.2) | 0 (0.0) | 2 (0.2) |  |  |
| Rivaroxaban 10 mg QD | 20 (1.7) | 1 (0.8) | 19 (1.8) |  |  |
| Rivaroxaban 10 mg BID | 3 (0.3) | 0 (0.0) | 3 (0.3) |  |  |
| Rivaroxaban 15 mg QD | 76 (6.4) | 7 (5.6) | 69 (6.5) |  |  |
| Rivaroxaban 15 mg BID | 29 (2.4) | 2 (1.6) | 27 (2.5) |  |  |
| Rivaroxaban 20 mg QD | 107 (9.0) | 7 (5.6) | 100 (9.4) |  |  |
| Aspirin with anticoagulant | 109 (9.1) | 12 (9.6) | 97 (9.1) | 0.979 | 0.018 |
| Clopidogrel with anticoagulant | 102 (8.5) | 7 (5.6) | 95 (8.9) | 0.281 | 0.127 |
| Ticagrelor with anticoagulant | 1 (0.1) | 0 (0.0) | 1 (0.1) | 1 | 0.043 |
| Anticoagulant with dual antiplatelet therapy | 143 (12.0) | 20 (16.0) | 123 (11.5) | 0.189 | 0.13 |
| **Imaging morphology of LVT** |  |  |  |  |  |
| LVEDD | 58.00 [53.00, 66.00] | 58.00 [52.00, 67.00] | 58.00 [53.00, 66.00] | 0.822 | 0.047 |
| LVEF | 38.00 [29.00, 46.00] | 40.00 [29.00, 47.00] | 38.00 [29.00, 46.00] | 0.433 | 0.065 |
| LVEF<=40% | 724 (60.7) | 70 (56.0) | 654 (61.2) |  | 0.106 |
| Global hypokinesis | 301 (25.2) | 22 (17.6) | 279 (26.1) | 0.049 | 0.207 |
| Hypokinesis | 516 (43.3) | 56 (44.8) | 460 (43.1) | 0.784 | 0.035 |
| Akinesis | 714 (59.8) | 76 (60.8) | 638 (59.7) | 0.894 | 0.022 |
| Apical LVT | 1080 (90.5) | 113 (90.4) | 967 (90.5) | 1 | 0.005 |
| Round LVT | 720 (60.4) | 68 (54.4) | 652 (61.0) | 0.18 | 0.135 |
| Mobile LVT | 97 (8.1) | 8 (6.4) | 89 (8.3) | 0.565 | 0.074 |
| Multiple LVT | 132 (11.1) | 12 (9.6) | 120 (11.2) | 0.688 | 0.054 |
| Calcified LVT | 214 (17.9) | 19 (15.2) | 195 (18.3) | 0.471 | 0.082 |
| LVT largest diameter/mm | 23.00 [17.00, 32.00] | 24.00 [19.00, 34.00] | 23.00 [17.00, 32.00] | 0.073 | 0.161 |
| LVT area/mm^2^ | 3.00 [1.65, 4.80] | 3.24 [1.84, 5.46] | 3.00 [1.64, 4.75] | 0.147 | 0.067 |
| Left ventricular aneurysm | 598 (50.1) | 64 (51.2) | 534 (50.0) | 0.873 | 0.024 |

Data are n/N (%), median (IQR) or mean (SD). SMD=standard mean difference. eGFR=estimated glomerular filtration rate. MI=myocardial infarction. CABG=coronary artery bypass grafting. PCI=percutaneous coronary intervention. STEMI=ST-segment elevation myocardial infarction. NSTEMI=non-ST-segment elevation myocardial infarction. ARVD=arrhythmogenic right ventricular dysplasia. NVM=noncompaction of the ventricular myocardium. DAPT=dual antiplatelet therapy. VKA=vitamin-K antagonists. DOAC=direct oral anticoagulants. LVEDD=left ventricular end diastolic dimension. LVEF=left ventricular ejection fraction. LVT=left ventricular thrombus.

**Supplemental Table 2. Baseline features according diabetes mellitus after propensity score matching.**

|  | Without diabetes | With diabetes | P value | SMD |
| --- | --- | --- | --- | --- |
| n | 382 | 382 |  |  |
| **Demographic** |  |  |  |  |
| Age/years | 55.79 (13.22) | 55.26 (13.40) | 0.581 | 0.04 |
| Male | 327 (85.6) | 323 (84.6) | 0.761 | 0.029 |
| Body mass index/kg/m^2^ | 25.32 [22.86, 27.76] | 25.09 [23.19, 27.65] | 0.877 | 0.011 |
| **Past medical history** |  |  |  |  |
| Hypertension | 203 (53.1) | 197 (51.6) | 0.717 | 0.031 |
| eGFR<60 ml/min/1.73m^2^ | 58 (15.2) | 63 (16.5) | 0.692 | 0.036 |
| Peripheral artery disease | 35 (9.2) | 35 (9.2) | 1 | <0.001 |
| Prior stroke | 60 (15.7) | 67 (17.5) | 0.56 | 0.049 |
| Prior MI | 220 (57.6) | 219 (57.3) | 1 | 0.005 |
| Prior CABG | 11 (2.9) | 11 (2.9) | 1 | <0.001 |
| Prior PCI | 64 (16.8) | 70 (18.3) | 0.634 | 0.041 |
| Prior cerebral hemorrhage | 3 (0.8) | 3 (0.8) | 1 | <0.001 |
| Atrial fibrillation | 35 (9.2) | 40 (10.5) | 0.627 | 0.044 |
| **Underlying disease** |  |  |  |  |
| Coronary artery disease | 307 (80.4) | 302 (79.1) | 0.719 | 0.033 |
| STEMI | 80 (20.9) | 72 (18.8) | 0.526 | 0.052 |
| NSTEMI | 15 (3.9) | 16 (4.2) | 1 | 0.013 |
| Dilated cardiomyopathy | 63 (16.5) | 63 (16.5) | 1 | <0.001 |
| Hypertrophic cardiomyopathy | 7 (1.8) | 8 (2.1) | 1 | 0.019 |
| ARVD with associated LV impairment | 0 (0.0) | 1 (0.3) | 1 | 0.072 |
| Perinatal cardiomyopathy | 1 (0.3) | 2 (0.5) | 1 | 0.042 |
| Restrictive cardiomyopathy | 3 (0.8) | 2 (0.5) | 1 | 0.032 |
| Alcoholic cardiomyopathy | 3 (0.8) | 2 (0.5) | 1 | 0.032 |
| Myocarditis | 3 (0.8) | 2 (0.5) | 1 | 0.032 |
| NVM | 8 (2.1) | 7 (1.8) | 1 | 0.019 |
| **Medications** |  |  |  |  |
| Aspirin | 226 (59.2) | 217 (56.8) | 0.558 | 0.048 |
| Clopidogrel | 196 (51.3) | 184 (48.2) | 0.426 | 0.063 |
| Ticagrelor | 16 (4.2) | 14 (3.7) | 0.852 | 0.027 |
| DAPT | 168 (44.0) | 155 (40.6) | 0.379 | 0.069 |
| VKA | 129 (33.8) | 135 (35.3) | 0.704 | 0.033 |
| Rivaroxaban | 82 (21.5) | 85 (22.3) | 0.861 | 0.019 |
| Dabigatran | 11 (2.9) | 8 (2.1) | 0.642 | 0.05 |
| DOAC | 93 (24.3) | 93 (24.3) | 1 | <0.001 |
| Antiplatelet therapy only | 149 (39.0) | 141 (36.9) | 0.602 | 0.043 |
| Anticoagulation only | 101 (26.4) | 109 (28.5) | 0.571 | 0.047 |
| Aspirin with anticoagulant | 36 (9.4) | 38 (9.9) | 0.903 | 0.018 |
| Clopidogrel with anticoagulant | 40 (10.5) | 39 (10.2) | 1 | 0.009 |
| Ticagrelor with anticoagulant | 382 (100.0) | 382 (100.0) | NA | <0.001 |
| Anticoagulant with dual antiplatelet therapy | 45 (11.8) | 42 (11.0) | 0.82 | 0.025 |
| **Imaging morphology of LVT** |  |  |  |  |
| LVEDD | 58.00 [52.00, 66.00] | 58.00 [54.00, 66.00] | 0.301 | 0.037 |
| LVEF | 39.00 [30.00, 45.00] | 37.00 [29.00, 45.83] | 0.495 | 0.041 |
| LVEF<=40% | 233 (61.0) | 237 (62.0) | 0.823 | 0.022 |
| Global hypokinesis | 89 (23.3) | 95 (24.9) | 0.672 | 0.037 |
| Hypokinesis | 182 (47.6) | 175 (45.8) | 0.664 | 0.037 |
| Akinesis | 231 (60.5) | 231 (60.5) | 1 | <0.001 |
| Apical LVT | 348 (91.1) | 347 (90.8) | 1 | 0.009 |
| Round LVT | 235 (61.5) | 229 (59.9) | 0.711 | 0.032 |
| Mobile LVT | 29 (7.6) | 33 (8.6) | 0.691 | 0.038 |
| Multiple LVT | 42 (11.0) | 45 (11.8) | 0.82 | 0.025 |
| Calcified LVT | 74 (19.4) | 73 (19.1) | 1 | 0.007 |
| LVT largest diameter/mm | 22.00 [17.00, 31.00] | 23.00 [16.00, 32.75] | 0.425 | 0.034 |
| LVT area/mm^2^ | 2.86 [1.61, 4.32] | 3.00 [1.62, 4.96] | 0.544 | 0.011 |
| Left ventricular aneurysm | 200 (52.4) | 193 (50.5) | 0.664 | 0.037 |

Data are n/N (%), median (IQR) or mean (SD). eGFR=estimated glomerular filtration rate. MI=myocardial infarction. CABG=coronary artery bypass grafting. PCI=percutaneous coronary intervention. STEMI=ST-segment elevation myocardial infarction. NSTEMI=non-ST-segment elevation myocardial infarction. ARVD=arrhythmogenic right ventricular dysplasia. NVM=noncompaction of the ventricular myocardium. DAPT=dual antiplatelet therapy. VKA=vitamin-K antagonists. DOAC=direct oral anticoagulants. LVT=left ventricular thrombus. LVEDD=left ventricular end diastolic dimension. LVEF=left ventricular ejection fraction. LVA=left ventricle aneurysms.

**Supplemental Table 3. Baseline features according diabetes mellitus after inverse probability of treatment weighting.**

|  | Without diabetes | With diabetes | P value | SMD |
| --- | --- | --- | --- | --- |
| n | 636.52 | 422.17 |  |  |
| **Demographic** |  |  |  |  |
| Age/years | 53.02 (15.20) | 53.71 (13.86) | 0.479 | 0.048 |
| Male | 532.1 (83.6) | 350.5 (83.0) | 0.823 | 0.015 |
| Body mass index/kg/m^2^ | 25.05 [22.49, 27.45] | 25.00 [23.03, 27.47] | 0.758 | 0.028 |
| **Past medical history** |  |  |  |  |
| Hypertension | 304.6 (47.8) | 211.8 (50.2) | 0.489 | 0.046 |
| eGFR<60 ml/min/1.73m^2^ | 92.0 (14.5) | 63.4 (15.0) | 0.806 | 0.016 |
| Peripheral artery disease | 48.2 (7.6) | 34.0 (8.1) | 0.785 | 0.018 |
| Prior stroke | 96.0 (15.1) | 66.1 (15.7) | 0.805 | 0.016 |
| Prior MI | 336.8 (52.9) | 223.7 (53.0) | 0.984 | 0.001 |
| Prior CABG | 13.9 (2.2) | 11.0 (2.6) | 0.692 | 0.028 |
| Prior PCI | 97.8 (15.4) | 65.0 (15.4) | 0.99 | 0.001 |
| Prior cerebral hemorrhage | 4.0 (0.6) | 2.4 (0.6) | 0.902 | 0.007 |
| Atrial fibrillation | 53.5 (8.4) | 37.6 (8.9) | 0.782 | 0.018 |
| **Underlying disease** |  |  |  |  |
| Coronary artery disease | 480.8 (75.5) | 322.0 (76.3) | 0.803 | 0.017 |
| STEMI | 135.8 (21.3) | 90.0 (21.3) | 0.995 | <0.001 |
| NSTEMI | 26.0 (4.1) | 17.6 (4.2) | 0.945 | 0.004 |
| Dilated cardiomyopathy | 104.9 (16.5) | 69.3 (16.4) | 0.976 | 0.002 |
| Hypertrophic cardiomyopathy | 15.8 (2.5) | 12.1 (2.9) | 0.756 | 0.023 |
| ARVD with associated LV impairment | 4.0 (0.6) | 0.9 (0.2) | 0.311 | 0.062 |
| Perinatal cardiomyopathy | 8.9 (1.4) | 5.2 (1.2) | 0.871 | 0.015 |
| Restrictive cardiomyopathy | 3.0 (0.5) | 2.2 (0.5) | 0.927 | 0.006 |
| Alcoholic cardiomyopathy | 8.1 (1.3) | 4.8 (1.1) | 0.873 | 0.012 |
| Myocarditis | 4.8 (0.8) | 3.2 (0.8) | 0.996 | <0.001 |
| NVM | 15.6 (2.5) | 9.7 (2.3) | 0.879 | 0.01 |
| **Medications** |  |  |  |  |
| Aspirin | 361.0 (56.7) | 237.9 (56.3) | 0.913 | 0.007 |
| Clopidogrel | 302.1 (47.5) | 201.4 (47.7) | 0.942 | 0.005 |
| Ticagrelor | 23.5 (3.7) | 13.8 (3.3) | 0.722 | 0.023 |
| DAPT | 262.3 (41.2) | 172.6 (40.9) | 0.922 | 0.007 |
| VKA | 224.9 (35.3) | 147.8 (35.0) | 0.923 | 0.007 |
| Rivaroxaban | 139.4 (21.9) | 95.1 (22.5) | 0.821 | 0.015 |
| Dabigatran | 14.8 (2.3) | 11.9 (2.8) | 0.693 | 0.031 |
| DOAC | 154.8 (24.3) | 107.0 (25.3) | 0.728 | 0.024 |
| Antiplatelet therapy only | 234.2 (36.8) | 152.6 (36.2) | 0.841 | 0.013 |
| Anticoagulation only | 189.6 (29.8) | 126.9 (30.1) | 0.929 | 0.006 |
| Aspirin with anticoagulant | 59.3 (9.3) | 40.9 (9.7) | 0.851 | 0.013 |
| Clopidogrel with anticoagulant | 56.9 (8.9) | 38.3 (9.1) | 0.944 | 0.005 |
| Ticagrelor with anticoagulant | 0.6 (0.1) | 0.0 (0.0) | 0.416 | 0.043 |
| Anticoagulant with dual antiplatelet therapy | 73.3 (11.5) | 48.7 (11.5) | 0.993 | 0.001 |
| **Imaging morphology of LVT** |  |  |  |  |
| LVEDD | 58.00 [52.00, 66.00] | 58.00 [54.00, 66.00] | 0.583 | 0.019 |
| LVEF | 38.00 [29.00, 45.94] | 37.00 [29.00, 46.00] | 0.807 | 0.008 |
| LVEF<=40% | 388.0 (61.0) | 259.1 (61.4) | 0.898 | 0.009 |
| Global hypokinesis | 162.9 (25.6) | 108.8 (25.8) | 0.953 | 0.004 |
| Hypokinesis | 279.6 (43.9) | 188.1 (44.6) | 0.847 | 0.013 |
| Akinesis | 384.5 (60.4) | 255.8 (60.6) | 0.956 | 0.004 |
| Apical LVT | 580.4 (91.2) | 382.2 (90.5) | 0.721 | 0.023 |
| Round LVT | 383.8 (60.3) | 254.7 (60.3) | 0.993 | 0.001 |
| Mobile LVT | 50.2 (7.9) | 32.9 (7.8) | 0.958 | 0.003 |
| Multiple LVT | 72.6 (11.4) | 51.4 (12.2) | 0.726 | 0.024 |
| Calcified LVT | 115.5 (18.1) | 78.3 (18.6) | 0.874 | 0.011 |
| LVT largest diameter/mm | 23.00 [17.00, 31.00] | 23.00 [16.00, 33.00] | 0.719 | 0.01 |
| LVT area/mm^2^ | 3.00 [1.70, 4.60] | 3.12 [1.62, 4.96] | 0.809 | 0.012 |
| Left ventricular aneurysm | 322.7 (50.7) | 217.8 (51.6) | 0.793 | 0.018 |

Data are n/N (%), median (IQR) or mean (SD). eGFR=estimated glomerular filtration rate. MI=myocardial infarction. CABG=coronary artery bypass grafting. PCI=percutaneous coronary intervention. STEMI=ST-segment elevation myocardial infarction. NSTEMI=non-ST-segment elevation myocardial infarction. ARVD=arrhythmogenic right ventricular dysplasia. NVM=noncompaction of the ventricular myocardium. DAPT=dual antiplatelet therapy. VKA=vitamin-K antagonists. DOAC=direct oral anticoagulants. LVT=left ventricular thrombus. LVEDD=left ventricular end diastolic dimension. LVEF=left ventricular ejection fraction. LVA=left ventricle aneurysms.

**Supplemental Table 4 Variance inflation factor for variables in the multivariable analysis.**

|  | **Age** | **Male** | **Diabetes mellitus** | **Body mass index/kg/m^2^** | **eGFR<60 ml/min/1.73m^2^** | **Hypertension** | **Prior stroke** | **Prior MI** | **Atrial fibrillation** | **LVEF<=40%** |
| --- | --- | --- | --- | --- | --- | --- | --- | --- | --- | --- |
| **VIF** | 1.391009 | 1.064294 | 1.0883 | 1.101583 | 1.131783 | 1.150756 | 1.056553 | 1.215559 | 1.044131 | 1.096323 |

VIF=Variance inflation factor. eGFR=estimated glomerular filtration rate. MI=myocardial infarction. LVEF=left ventricular ejection fraction.

**Supplemental Table 5 Correlation matrix for variables in the multivariable analysis.**

|  | **Age** | **Male** | **Diabetes mellitus** | **Body mass index/kg/m^2^** | **eGFR<60 ml/min/1.73m^2^** | **Hypertension** | **Prior stroke** | **Prior MI** | **Atrial fibrillation** | **LVEF<=40%** |
| --- | --- | --- | --- | --- | --- | --- | --- | --- | --- | --- |
| **Age** | 1 | 0.01 | 0.19 | -0.13 | 0.25 | 0.21 | 0.17 | 0.38 | 0.14 | -0.16 |
| **Male** | 0.01 | 1 | -0.06 | 0.14 | -0.13 | 0.06 | 0 | 0.12 | -0.01 | -0.09 |
| **Diabetes mellitus** | 0.19 | -0.06 | 1 | 0.06 | 0.16 | 0.12 | 0.1 | 0.08 | 0.12 | 0.06 |
| **Body mass index/kg/m^2^** | -0.13 | 0.14 | 0.06 | 1 | -0.03 | 0.18 | -0.05 | -0.05 | 0.01 | -0.04 |
| **eGFR<60 ml/min/1.73m^2^** | 0.25 | -0.13 | 0.16 | -0.03 | 1 | 0.12 | 0.06 | 0.07 | 0.08 | 0.12 |
| **Hypertension** | 0.21 | 0.06 | 0.12 | 0.18 | 0.12 | 1 | 0.16 | 0.14 | -0.03 | -0.13 |
| **Prior stroke** | 0.17 | 0 | 0.1 | -0.05 | 0.06 | 0.16 | 1 | 0.09 | 0.06 | 0.01 |
| **Prior MI** | 0.38 | 0.12 | 0.08 | -0.05 | 0.07 | 0.14 | 0.09 | 1 | -0.01 | -0.17 |
| **Atrial fibrillation** | 0.14 | -0.01 | 0.12 | 0.01 | 0.08 | -0.03 | 0.06 | -0.01 | 1 | 0.01 |
| **LVEF<=40%** | -0.16 | -0.09 | 0.06 | -0.04 | 0.12 | -0.13 | 0.01 | -0.17 | 0.01 | 1 |

eGFR=estimated glomerular filtration rate. MI=myocardial infarction. LVEF=left ventricular ejection fraction.

**Supplemental Table 6 Multivariable analysis for the relationship between DOAC and adverse outcomes in LVT patients receiving anticoagulation.**

|  | **In LVT patients with anticoagulation**  **(n=638)** | | **In non-diabetic LVT patients with anticoagulation** | | **In diabetic LVT patients with anticoagulation** | |  |
| --- | --- | --- | --- | --- | --- | --- | --- |
| **Endpoints** | **Hazard ratio (95%CI)** | **P values** | **Hazard ratio (95%CI)** | **P values** | **Hazard ratio (95%CI)** | **P values** | **P for interaction** |
| **All-cause death** | 1.40 (0.90-2.18) | 0.14 | 1.16 (0.58-2.29) | 0.7 | 1.64 (0.91-2.95) | 0.10 | 0.13 |
| **Cardiovascular death** | 1.25 (0.79-1.96) | 0.3 | 0.83 (0.39-1.74) | 0.6 | 1.63 (0.91-2.92) | 0.1 | 0.054 |
| **MACCE** | 1.30 (0.86-1.96) | 0.2 | 0.85 (0.43-1.68) | 0.6 | 1.73 (1.03-2.92) | 0.038 | 0.022 |

LVT=left ventricular thrombus. DOAC=direct oral anticoagulants. MACCE=major adverse cardiac and cerebrovascular events. CI=confidence interval.

The presented hazard ratio is multivariable adjusted for age, gender, eGFR<60ml/min/1.73m^2^, body mass index, ejection fraction<=40%, hypertension, prior myocardial infarction, prior stroke, and atrial fibrillation.
